# Editorial: The Role of Resilience and the Interplay Between Genetics and Environment in Bipolar Disorder

**DOI:** 10.3389/fpsyt.2021.761384

**Published:** 2021-11-19

**Authors:** Bianca Pfaffenseller, Thiago Wendt Viola, Adriane R. Rosa, Gabriel R. Fries

**Affiliations:** ^1^Department of Psychiatry and Behavioural Neurosciences, McMaster University, Hamilton, ON, Canada; ^2^Developmental Cognitive Neuroscience Lab, School of Medicine, Pontifical University Catholic of Rio Grande do Sul (PUCRS), Porto Alegre, Brazil; ^3^Department of Pharmacology, Federal University of Rio Grande do Sul, Porto Alegre, Brazil; ^4^Laboratory of Molecular Psychiatry, Clinic Hospital of Porto Alegre, Porto Alegre, Brazil; ^5^Translational Psychiatry Program, Faillace Department of Psychiatry and Behavioral Sciences, The University of Texas Health Science Center at Houston, Houston, TX, United States

**Keywords:** bipolar disorder, resilience, illness course, stress, depression

There is a major need to understand why a subset of patients with bipolar disorder may develop a progressive course related to distinct neurobiological underpinnings (1) and worse clinical outcomes, such as reduced responsiveness to treatment, and impaired cognition and functioning (2, 3). Understanding why different subjects show varying degrees of risk may provide valuable information to the field, ultimately informing mechanisms related to heterogeneous clinical presentations. Resilience may be a key factor in this process. Resilience refers to the ability to adapt well in the face of adversity, trauma, or stress, contributing to maintain normal psychological and physical functioning (4, 5). Resilience has been a relevant topic in the field of major psychiatric disorders (6, 7); however, research on the role of resilience in bipolar disorder is still scarce. The present Research Topic on “*The Role of Resilience and the Interplay between Genetics and Environment in Bipolar Disorder*” makes a contribution with original research articles and a review.

There are different ways in understanding, measuring, and studying resilience in *in vitro*, animal, and human studies (6). Within this Research Topic, two articles considered resilience in the context of factors that could negatively impact the illness course in bipolar disorder, including stressful situations in life (Carta et al.) and the self-stigma (Post et al.), when patients apply negative stereotypes stigmatizing attitudes toward themselves. As a potential protective factor, resilience can have a positive impact on the long-term outcomes in bipolar disorder, which could include a positive role in promoting stress management (Carta et al.) and stigma resistance (Post et al.). In this context, Post et al. investigated the association between resilience, premorbid functioning, and residual mood symptoms, with self-stigma and stigma resistance in stabilized patients. They found that high resilience is associated with low self-stigma and higher stigma resistance, while residual depressive symptoms were correlated positively with self-stigma and negatively with stigma resistance. In addition, they showed that low resilience was a predictor of higher self-stigma and high resilience of higher stigma resistance. Therefore, resilience may be an important factor in preventive and therapeutic strategies to protect from self-stigma and promote stigma resistance in patients with bipolar disorder. Considering that stressful events in life could have an important impact on individuals, communities, and societies, promoting resilience has become a major need at this moment when people worldwide are under considerable stress due to the COVID-19 pandemic. The COVID-19 pandemic and subsequent lockdown has produced drastic changes and consequences for people's mental health. In this sense, Carta et al. aimed to investigate whether restrictions during COVID-19 pandemic lockdown could influence the clinical course of bipolar disorder. This longitudinal exploratory study was conducted with two samples in Europe (clinically stable before the lockdown and living in two geographically close cities: one with a rigid lockdown and another with a less severe one). The authors showed that patients with bipolar disorder in the region with more severe lockdown restrictions reported clinically relevant depressive symptoms and more dysfunctional scores in the areas of sleep, activities, and social rhythms. Therefore, a rigid lockdown could expose patients with bipolar disorder to depressive relapse and dysregulation of biological rhythms. In this scenario, public health strategies promoting resilience have become a priority.

Other two articles in this Research Topic discuss molecular mechanisms that are potentially associated with resilience. Considering that different individuals respond to stress differently because of resilience, Song and Wang obtained resilience mice from chronic unpredictable mild stress (CUMS) and a companion group (a reward condition, mimicking a partner or close friend) to study mechanisms involved in the improvement of stress-induced behaviors and to detect associated molecular profiles. Their results showed that CUMS-induced depressive-like behavior was ameliorated by the presence of a companion, which reinforces evidence in patients that social support is associated with depression recovery. In both depressive-like behavior and resilient mice, there were several differentially expressed genes in the ventral tegmental area as compared to controls, which were found to be mainly enriched in synapse, neuronal cell body, axon, and transport vesicles, and involved in signal transduction and signaling molecules and interactions. This study provides theoretical support for reward intervention in the treatment of depression and suggests potential drug targets. Also exploring novel targets for treatment, the study by Gonzalez discusses the mitonuclear incompatibility in bipolar disorder, an interesting mechanism by which mitochondria and the nucleus crosstalk with each other to allow for their genomes to remain compatible. In other words, a mutation in the mitochondrial DNA (mtDNA) typically activates an alteration in the nuclear genome to off-set or compensate for the mtDNA change, ultimately preventing mitochondrial dysfunction and its consequences to the cell. While this mitonuclear interaction may have taken place through many years of evolution, their disconnections (for instance, in admixed populations, such as Hispanic and African Americans, where mtDNA and nuclear genomes may have different origins) could lead to mitochondrial dysfunctions, which have been strongly associated with the pathophysiology of bipolar disorder. Thus, the review by Gonzalez cites evidence of higher prevalence of severe mental illness in adults reporting two or more races and discusses how this mechanism may be key to the mitochondrial dysfunction reported for bipolar disorder and resilience against environmental stressors.

In summary, this Research Topic addresses resilience from different perspectives, bringing together findings focused on factors that could impact the illness course in bipolar disorder and exploring molecular mechanisms that are potentially associated with resilience. Several studies have already contributed to understanding the biological and clinical changes associated with bipolar disorder; however, more dedicated research on the role of resilience is needed to advance the current knowledge in the field. A coordinated effort involving research from different perspectives including a variety of disciplines, such as psychology, psychiatry, neurobiology, pharmacology, and basic science studies, will be crucial to provide a clearer understanding about the role of resilience in bipolar disorder. In addition to interdisciplinary studies, longitudinal research designs are needed to investigate the mechanisms of resilience, which could involve the interplay between genetics and environment. That could contribute to elucidating the factors influencing the risk of developing bipolar disorder and worse outcomes, help identify vulnerable patients, and thus provide novel insights into personalized medicine centered on therapeutic interventions and prevention strategies.

## Author Contributions

BP conceptualized the article. BP, TW, AR, and GF co-wrote the article. All authors revised the article and approved the submitted version.

## Funding

AR thanks to the CNPq process 302382-2019-4. GF was funded by the National Institute of Mental Health (NIMH, 5K01MH121580) and by the American Foundation for Suicide Prevention (AFSP, YIG-0-066-20).

## Conflict of Interest

The authors declare that the research was conducted in the absence of any commercial or financial relationships that could be construed as a potential conflict of interest.

## Publisher's Note

All claims expressed in this article are solely those of the authors and do not necessarily represent those of their affiliated organizations, or those of the publisher, the editors and the reviewers. Any product that may be evaluated in this article, or claim that may be made by its manufacturer, is not guaranteed or endorsed by the publisher.
